# Evaluation of bactericidal potential and catalytic dye degradation of multiple morphology based chitosan/polyvinylpyrrolidone-doped bismuth oxide nanostructures

**DOI:** 10.1039/d2na00105e

**Published:** 2022-05-04

**Authors:** Ahsaan Bari, Muhammad Ikram, Ali Haider, Anwar Ul-Hamid, Junaid Haider, Iram Shahzadi, Ghazanfar Nazir, Anum Shahzadi, M. Imran, Abdul Ghaffar

**Affiliations:** Solar Cell Applications Research Lab, Department of Physics, Government College, University Lahore Lahore 54000 Punjab Pakistan dr.muhammadikram@gcu.edu.pk; Department of Clinical Sciences, Faculty of Veterinary and Animal Sciences, Muhammad Nawaz Shareef, University of Agriculture (MNSUA) 66000 Punjab Pakistan; Core Research Facilities, King Fahd University of Petroleum & Minerals Dhahran 31261 Saudi Arabia anwar@kfupm.edu.sa; Tianjin Institute of Industrial Biotechnology, Chinese Academy of Sciences Tianjin 300308 China; Punjab University College of Pharmacy, University of the Punjab Lahore 54000 Pakistan; Department of Nanotechnology and Advanced Materials Engineering, Sejong University Seoul 05006 Republic of Korea; Faculty of Pharmacy, University of the Lahore Lahore Pakistan; Department of Chemistry, Government College University Faisalabad Pakpattan Road Sahiwal Punjab 57000 Pakistan; Department of Physics, Government College University Lahore 54000 Pakistan

## Abstract

In this study, 0.02 and 0.04 wt% of chitosan (CS) were successfully incorporated in a fixed amount of polyvinylpyrrolidone (PVP)-doped Bi_2_O_3_ nanostructures (NSs) *via* a co-precipitation approach. The purpose of this research was to degrade hazardous methylene blue dye and assess antimicrobial potential of the prepared CS/PVP-doped Bi_2_O_3_ nanostructures. In addition, optical characteristics, charge recombination rate, elemental composition, phase formation, surface morphology, functional groups, *d*-spacing, and crystallinity of the obtained nanostructures were investigated. CS/PVP-doped Bi_2_O_3_ nanostructures exhibited efficient catalytic activity (measured as 99%) in a neutral medium for dopant-free nanostructures while the inhibition zone was measured using a Vernier caliper against pathogens *Escherichia coli* (*E. coli*) and *Staphylococcus aureus* (*S. aureus*) at low and high doses to check antimicrobial activity. Strong bactericidal action was recorded against *S. aureus* bacteria such that a significant inhibition zone was measured at 3.09 mm.

## Abstract 2

In this study, 0.02 and 0.04 wt% of chitosan (CS) were successfully incorporated in a fixed amount of polyvinylpyrrolidone (PVP)-doped Bi_2_O_3_ nanostructures (NSs) *via* a co-precipitation approach.

## Graphical abstract

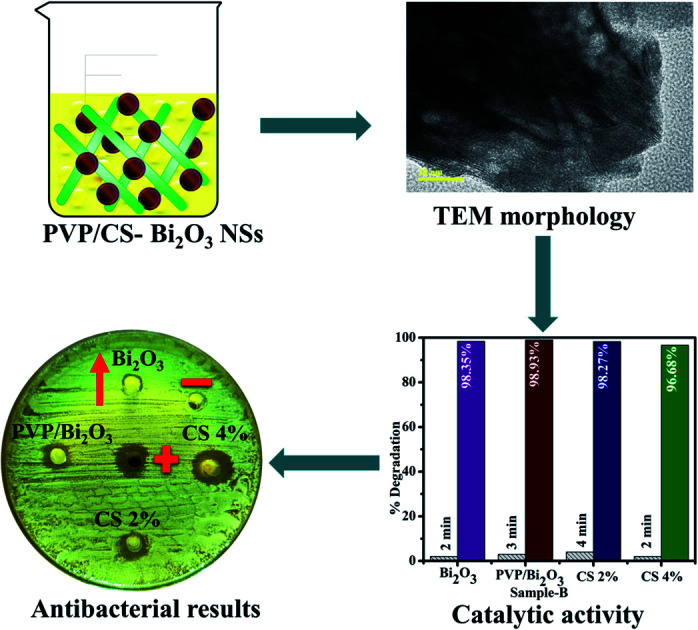

## 1 Introduction

Increased economic activity and fast industrial growth have exacerbated water pollution and health-related concerns globally.^1^ World Health Organization (WHO) estimates that each year 2.3 million people die from water-borne (typhoid, cholera, hepatitis, and diarrhea) and carcinogenic diseases.^2–4^ Around 70% of water pollution is produced due to industrial waste dyes (acidic, basic, and azoic) and heavy metals (cadmium, chromium, nickel, lead, *etc.*). All such pollutants are highly soluble in nature and pose a serious health risk to humans and wildlife.^5^ Dyes are excessively used in paper coloring *i.e.*, temporary hair colorants, cotton dyeing, and paper stock coating. Especially methylene blue (MB), a basic dye, has an aromatic molecular structure that is stable and nonbiodegradable posing strong ecological threat to aquatic life.^6^ According to published research, 15% of the most extensively produced dyes are released into water bodies both directly and indirectly.^7^ As a result, it is important to employ a method capable of degrading synthetic dye directly into non-toxic molecules such as water and carbon dioxide. Scientists use traditional approaches such as chlorination, aerobic treatment, adsorption, and ion exchange to remove organic contaminants from water. Unfortunately, these techniques have drawbacks and limitations such as high energy consumption, secondary pollution caused by inadequate removal, and transfer of dye.^8,9^ Catalysis in the presence of nanomaterial-based semiconductors attracted interest of researchers owing to their minimal toxicity, chemical stability, low cost and nature-friendly characteristics.^10^ In order to degrade synthetic dyes such as MB, this research uses a reducing agent and nano-catalyst.^11–13^ Mastitis has a substantial economic burden on the dairy sector. Infectious agents such as bacteria, viruses, and fungi cause mastitis. Chemical, microbial, and physical changes in milk, and clinical abnormalities in mammary gland tissues, are all associated with this disease.^14^*Coliform*, *Escherichia coli* and *Staphylococcus aureus* are the most prevalent bacterial pathogens linked to mastitis.^15,16^

Nanomaterials have attracted researcher's attention due to their unique physiochemical properties and enhanced dye-contaminated wastewater treatment methods.^17–19^ Small NSs with size ranging from 1 to 100 nm have astonishing surface-to-volume ratios when compared to those of bulk chemical compositions, resulting in significant increases in chemical (biological, catalytic activity, *etc.*) and physical properties. Metal oxide nanomaterials have large surface area, and attractive nanostructural, optical, mechanical, and thermodynamic characteristics that are advantageous for catalysis and antibacterial activities.^20^ Numerous metal oxide nanomaterials (ZnO, TiO_2_, La_2_O_3_, CeO_2_, and Bi_2_O_3_) are being used in catalysis and to check antibacterial activity; particularly as an important p-type semiconductor, Bi_2_O_3_ has remarkable anode semiconductor properties including a broad band gap, low toxicity, high conductivity, antibacterial activity and degradation capability for organic dyes.^21–29^ Chemical (co-precipitation, sol gel, and redox reactions) and green synthesis techniques are utilized to synthesize Bi_2_O_3_ NSs.^21,22,30,31^ Among these, the co-precipitation method is considered as ecofriendly, inexpensive, energy-efficient and easy to use.^23^ A number of research studies were conducted on Bi_2_O_3_ NSs prepared through various synthesis routes to check the influence of antimicrobial activity and dye degradation.^32–41^ However, the obtained results were not impressive for bactericidal action and dye degradation performance. The addition of a polymer into metal oxides increases their stability and improves physiochemical properties which results in efficient dye degradation and antibacterial performance.

Polymers can interact with metal ions either through complex or ion-pair formation, which might be an attractive substitute for a stabilizer and thus can be targeted to attain specific physicochemical parameters of NSs.^24^ Polymeric materials have received much attention from scientists for usage in biological and environmental applications.^42^ Numerous types of polymers (polyvinyl alcohol, polyvinyl chloride, polyvinylpyrrolidone, and chitosan) are used for metal oxide doping to attain significant outcomes for various applications.^43–45^ Among them, PVP is a synthetic polymer that is considered an effective capping agent for metal oxide NSs. Its properties are attributed to the presence of both carbonyl groups and functional groups that strengthen metal oxide NSs within its composite.^46,47^ As it exhibits excellent physicochemical properties, it is used as an additive in different materials and to stabilize NSs.^48–51^ Coincidently, recent studies have shown that PVP has great water solubility, low toxicity, biocompatibility, and exhibits promising results against antimicrobial activity.^52–55^ Chitosan is an alkaline polymer prepared by partially hydrolyzing chitin, the primary component of crustaceans and fungus cells, and extensively used for pharmaceutical and biomedical purposes. It has superior biodegradability, biocompatibility, low toxicity, and film-forming characteristics.^56^ CS is mainly composed of amino and hydroxyl groups, both significant in metal ion chemical adsorption, and these groups can bind with metal ions more efficiently than any other polysaccharide, making a strong template for synthesizing metal oxide NSs.^57–61^

The motivation of this research is to synthesize PVP/CS-doped Bi_2_O_3_ NSs utilizing an ecologically friendly co-precipitation technique for degradation of organic dyes from contaminated water and also to assess material's bactericidal potential. Numerous characterization techniques were employed for detailed analysis of synthesized NSs. Catalytic activity (CA) tests were performed for degradation of MB dye. Furthermore, *Staphylococcus aureus* (*S. aureus*) and *Escherichia coli* (*E. coli*) pathogens were used to examine its potential for antibacterial activity.

## 2 Experimental section

### 2.1 Materials

Bismuth nitrate (Bi(NO_3_)_3_·5H_2_O, 98%) was acquired from BDH Laboratory Supplies. Sodium hydroxide (NaOH, 98%), polyvinylpyrrolidone (PVP) and chitosan (CS) were supplied by Sigma-Aldrich.

### 2.2 Synthesis of polyvinylpyrrolidone/chitosan-doped bismuth oxide

**Fig. 1:**
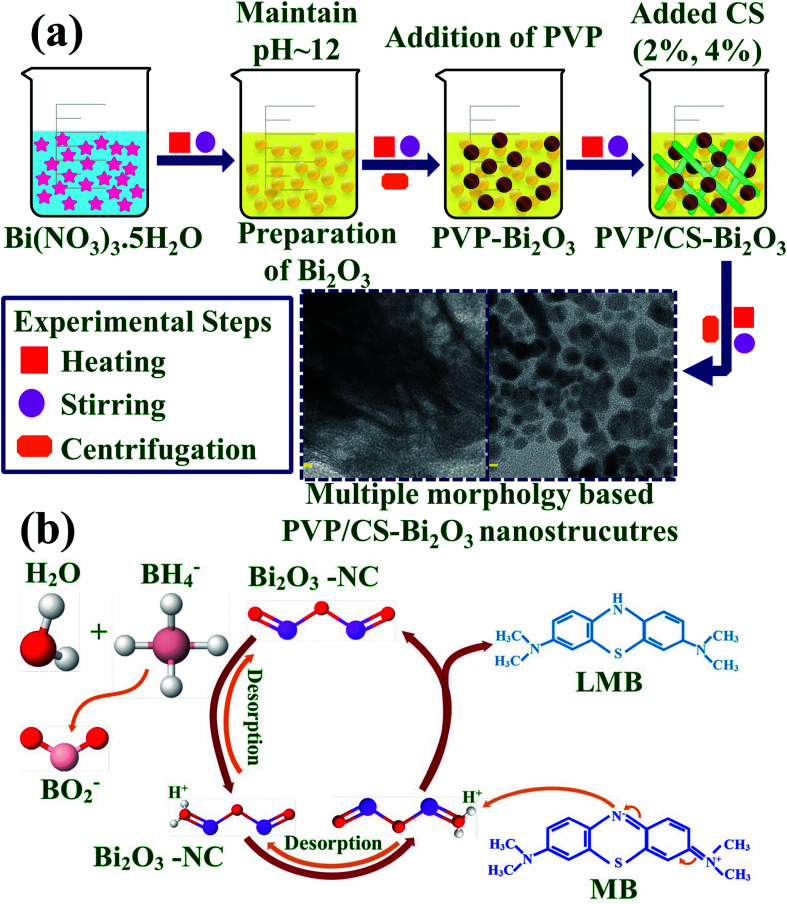
(a) Synthesis of PVP/CS-doped Bi_2_O_3_ (b) catalysis mechanism of the prepared NSs.

0.5 M of Bi_2_O_3_ was prepared under continuous stirring at 80 °C. NaOH was incorporated dropwise to maintain pH ≈ 12 under vigorous stirring for 1 hour. Further the colloidal solution was centrifuged at 7500 rpm repeatedly and dried for 12 hours at 150 °C. The obtained Bi_2_O_3_ NSs were crushed into fine powder. Using the above method, the desired amount of PVP was dissolved to prepare PVP-doped Bi_2_O_3_ and various concentrations of CS (2% and 4%) were added to get PVP/CS**-**doped Bi_2_O_3_ NSs as represented in Fig. 1(a).

### 2.3 Catalysis

The degradation efficiency of synthetic dyes in the presence of sodium borohydride (NaBH_4_) and the synthesized nano-catalyst was determined through CA measurements. MB is a positively charged thiazine dye frequently used as a reductant in analytical chemistry, and is colorless in the reduced form and blue in the oxidized form.^62^ Using a quartz cell, 0.1 M NaBH_4_ solution (400 μL) was dissolved in 3 ml MB. Furthermore, 400 μL synthesized NS solution was incorporated in aqueous solution of MB. Absorption reaction progress was spectrophotometrically monitored at room temperature. In the presence of NaBH_4_, MB changed to leucomethylene confirming degradation of dyes. Samples without a nano-catalyst were referred to as blank. % degradation was calculated as:
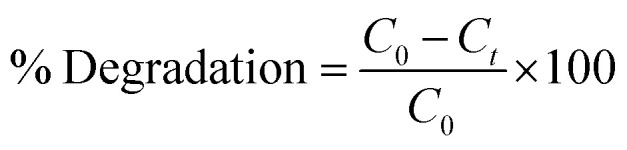
where *C*_0_ represents initial absorbance and *C*_*t*_ represents the concentration at specific time.

#### 2.3.1 Catalysis mechanism

Adding a nano-catalyst and reducing agent to the dye are the major factors considered to be significant in the catalysis mechanism as demonstrated in Fig. 1(b). The chemical material provides an e^−^ to the ongoing reaction referred to as the reducing agent. MB receives an e^−^ from the diminishing agent in a chemical reaction to act as an oxidizing agent. The redox reaction occurs during CA, and involves the transfer of an e^−^ from the reductant to the acceptor of an oxidant. This leads to electron absorption in MB and causes the breakdown of the synthetic dye. Furthermore, MB was tested in the presence of reducing agent (NaBH_4_), this oxidation reaction was incredibly slow and time consuming. To overcome these issues, incorporation of nano-catalysts (Bi_2_O_3_ and PVP/CS (2%, 4%)-doped Bi_2_O_3_) into oxidation–reduction reactions serves as electron relay and allows electron transfer from the donor (BH_4_^−^) to the acceptor (MB). Adsorption of BH_4_^−^ ions and dye molecules is increased by using NSs while a large number of active sites encourage them to react with each other quicker resulting in efficient dye degradation.^63,64^ The presence of a reducing agent and nano-catalyst increases the degradation efficiency. As reported above, a catalytic route was adopted for dye degradation utilizing reducing agents and nano-catalysts in this study.^65^

### 2.4 Isolation and identification of *Staphylococcus aureus* and *Escherichia coli*

Large quantities of dairy milk specimens were obtained from Pakistani public, private institutions and dairy farms and evaluated for surf-field mastitis. Furthermore, the acquired samples were incubated in 5% sheep blood agar. On Mannitol salt agar (MSA) and MacConkey agar (MA), colonies were formed in order to isolate Gram-positive (G +ve) *S. aureus* and Gram-negative (G −ve) *E. coli* pathogens, respectively. Pharmacological (catalase and coagulase) and morphological (gram staining) methodologies were used to identify distinctive colonies.

### 2.5 Antimicrobial activity

Antibacterial performance of the prepared NSs was examined through the agar well diffusion approach with germ strain (G +ve and G −ve) swabbed 1.5 × 10^8^ CFU mL^−1^ on MSA and MA for *S. aureus* and *E. coli*, respectively. Moreover, negative and positive controls were assigned to DIW (50 μL) and ciprofloxacin (0.005 mg/50 μL), correspondingly. Different concentrations of Bi_2_O_3_ and PVP/CS (2%, 4%)-doped Bi_2_O_3_ NSs were injected into a 6 mm well on MSA and MA plates with a micropipette and sterilized cork borer at low (0.5 mg/50 μL) and high (1.0 mg/50 μL) doses. Petri plates containing doses were incubated for 24 h at 37 °C. Furthermore, a Vernier caliper was used to assess the diameter of the inhibition zone that results in the determination of antibacterial performance. One-way variance analysis in SPSS 20 was employed to determine the bacterial efficiency by measuring the inhibitory zone.

### 2.6 Characterization techniques

Structural and crystalline behaviors of obtained powder were determined using powder XRD ranging from 10° to 60°. FTIR spectroscopy was performed between 4000 and 400 cm^−1^ to identify functional groups present in PVP/CS (2%, 4%)-doped Bi_2_O_3_ NSs. The chemical composition, surface study, morphology and *d*-spacing of PVP/CS (2%, 4%)-doped Bi_2_O_3_ NSs were analyzed through EDS, SEM and HR-TEM respectively. Additionally, SAED analysis was performed to check crystallinity of the prepared samples. A Genesys 10S UV-vis spectrophotometer was employed to determine the optical properties while PL spectroscopy was used to investigate electron–hole recombination in the synthesized sample.

## 3 Results and discussion

**Fig. 2:**
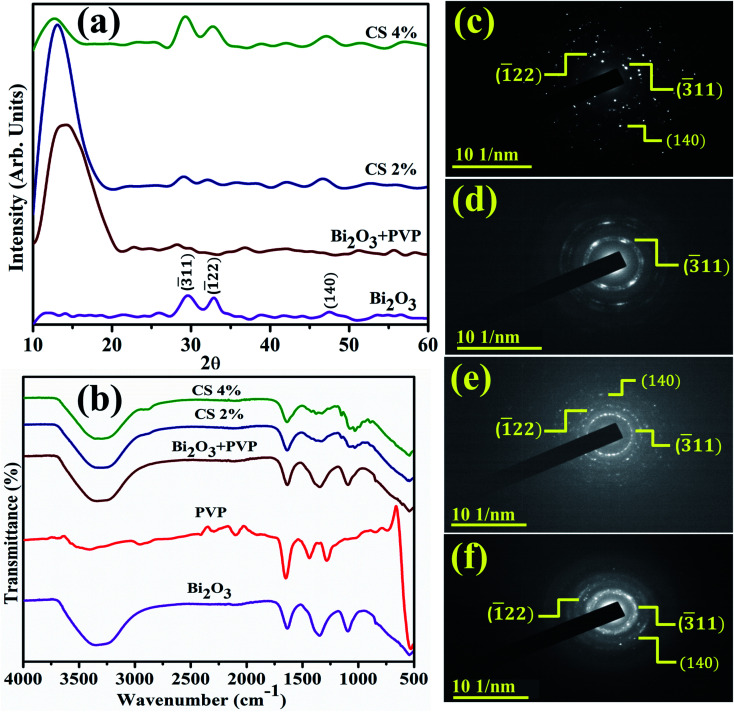
(a) XRD patterns, (b) FTIR spectra and (c–f) SAED images of PVP/CS-Bi_2_O_3_ (2% and 4%) NSs.

XRD identifies NSs crystallinity, crystal structure, and crystal size ranging from 2*θ* 10–60° (Fig. 2(a)). Diffraction peaks appearing at 2*θ* values 29.503° (3̄11), 33.040° (1̄22), and 47.577° (140) revealed the monoclinic structure of Bi_2_O_3_, well matched with the XRD standard card (JCPDF 01-083-0410/00-041-1449).^66^ An additional broad characteristic peak observed near 13.5° corresponds to PVP and shows its amorphous nature.^67^ Upon doping, PVP peaks become more broadened and less intense than that of a pure nanocatalyst owing to enhancement in structural instability and the decrease in the crystallite size.^68,69^ Furthermore, an increasing amount of CS results in diffraction peak shift while decreased intensity is attributed to the enhanced structural disorders and significant decrease in the crystallite size. The crystallite size calculated from the most intense peak of all prepared samples using the Debye–Scherrer formula was 69.5 nm, 17.5 nm, 58.4 nm, and 26.25 nm for Bi_2_O_3_ and PVP/CS (2%, 4%)-doped Bi_2_O_3_ NSs respectively. FTIR was used to elucidate functional groups in the prepared Bi_2_O_3_ NSs (Fig. 2(b)). The Bi–O–Bi stretching vibration, C–C stretching and product vibration mode of NO_3_ were assigned to 540 cm^−1^, 1076 cm^−1^, 1357 cm^−1^ bands correspondingly.^31,70,71^ The manifested band at 1640 cm^−1^ was attributed to the bending vibration of H_2_O while the band at 3300–3500 cm^−1^ corresponded to the stretching vibration of absorbed hydroxyl function groups.^72,73^ Furthermore, bands at 2950 cm^−1^ and 1652 cm^−1^ in FTIR spectra of PVP were assigned to the presence of asymmetric stretching of CH_2_ and stretching of C–O (amide C=O bond), respectively.^74^ Bands at 1423 cm^−1^, 1288 cm^−1^, and 1652 cm^−1^ have been assigned to C–H bending, CH_2_ wagging and the –C=O group respectively.^74^ With the addition of pure PVP in the prepared Bi_2_O_3_ NSs, the band which appeared at 1652 cm^−1^ shifted towards a lower wavenumber at 1640 cm^−1^ probably indicating that the C=O bond is getting weakened and there exists an interaction between metal ions and PVP through oxygen of the C=O group of the polymer. This distinguishing band may show the interaction of PVP with metal ions.^75^ Upon doping with chitosan, shift of bands toward a lower wavelength was observed, which is attributed to the –OH or NH_2_ functional group of CS.^76–78^ Strong hydrogen bonding interactions between two types of molecules form a homogeneous phase.^79^ Additionally, SAED analysis indicates bright circular rings of Bi_2_O_3_ and PVP/CS-Bi_2_O_3_ NSs represented in Fig. 2(c–f) suggesting highly crystalline nature of the samples. XRD measurements satisfying Bragg's diffraction conditions were well correlated with various planes of NSs.

**Fig. 3:**
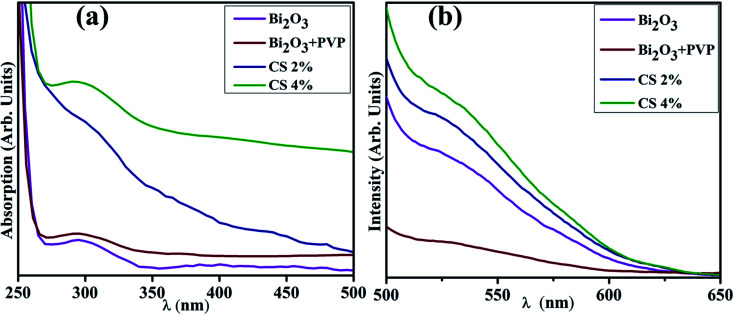
(a) UV-vis spectra (b) PL spectra of PVP/CS (2%, 4%)-doped Bi_2_O_3_ NSs.

Fig. 3(a) reveals the band gap energy (*E*_g_) and optical properties of the synthesized samples assessed with a UV-visible spectrophotometer between 250 and 500 nm. It shows a considerable absorption peak at 295 nm for Bi_2_O_3_.^80^ The wavelength acquired from UV-visible absorption spectra determined *E*_g_ of dopant free and PVP/CS-doped Bi_2_O_3_ NSs to be 4.18 eV, 4.27 eV, 4.09, and 4.13 eV respectively.^80^ Upon doping with PVP, the absorption in higher wavelength (blue shift) was observed, ascribed to an increase in *E*_g_ and decrease in the crystallite size. Furthermore, addition of CS resulted in absorption toward longer wavelength (red shift) indicating a decrease in *E*_g_ and increase in the crystallite size. Increasing amount of CS reduces the crystallite size that results in increased *E*_g_ which is well matched with the XRD results.

PL analysis elucidates the electron–hole pair recombination process in all synthesized samples as shown in Fig. 3(b). The photoluminescence signal is produced when electrons in the VB are excited to the CB at an excitation wavelength and subsequently return to the VB.^81^ Bi_2_O_3_ NSs emit broad emission peaks in the visible range from 520–542 nm, attributed to Bi^3+^ ions, when excited at 300 nm.^82^ The luminescence of ions in the green region is produced by the ^3^P_1_–^1^S_0_ transitions, or charge transfer between the bonding oxygen and Bi^3+^ ions.^83–85^ When PVP was incorporated, peak intensity decreased, indicating lower charge recombination while peak intensity increased upon increasing the concentration of CS, which suggests a high photo-generated charge carrier recombination tendency.^86^

**Fig. 4:**
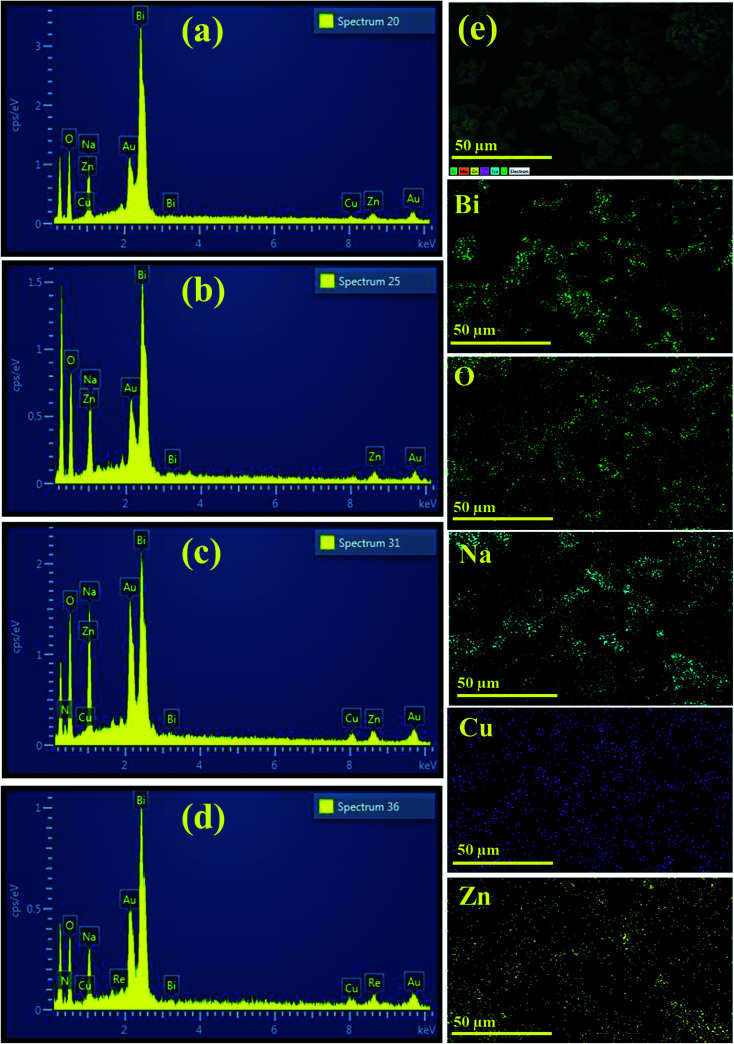
EDS image of (a) Bi_2_O_3_ (b) PVP-doped Bi_2_O_3_ (c) PVP/CS 2%-Bi_2_O_3_ (d) PVP/CS 4%-Bi_2_O_3_ and (e) mapping image of PVP/CS 4%-Bi_2_O_3_ NSs.

The chemical composition of PVP/CS (2%, 4%)-doped Bi_2_O_3_ NSs determined through EDS is represented in Fig. 4(a–d). Strong peaks of Bi and O were observed that confirm the presence of Bi_2_O_3_ NSs in the synthesized samples. The carbon peak is attributed to PVP/CS used in the samples. The sodium (Na) peak was probably generated by the use of NaOH to sustain the pH of samples while Au peaks originate due to the coating sputtered upon the samples to reduce charging effects. Small peaks of Cu and Zn could be attributed to the effect of the brass sample holder utilized during EDS observation and to some contamination. Additionally, EDS mapping of the as-prepared higher doped specimen was carried out to analyze the distribution pattern of its elemental constituents in order to check additional interfacial contact as represented in (e). Five components (Bi, O, Na, Cu, and Zn) were found to spread in the higher doped samples. As already mentioned, Na, Cu, and Zn were assigned to contamination, the sample holder used for EDS analysis.

**Fig. 5:**
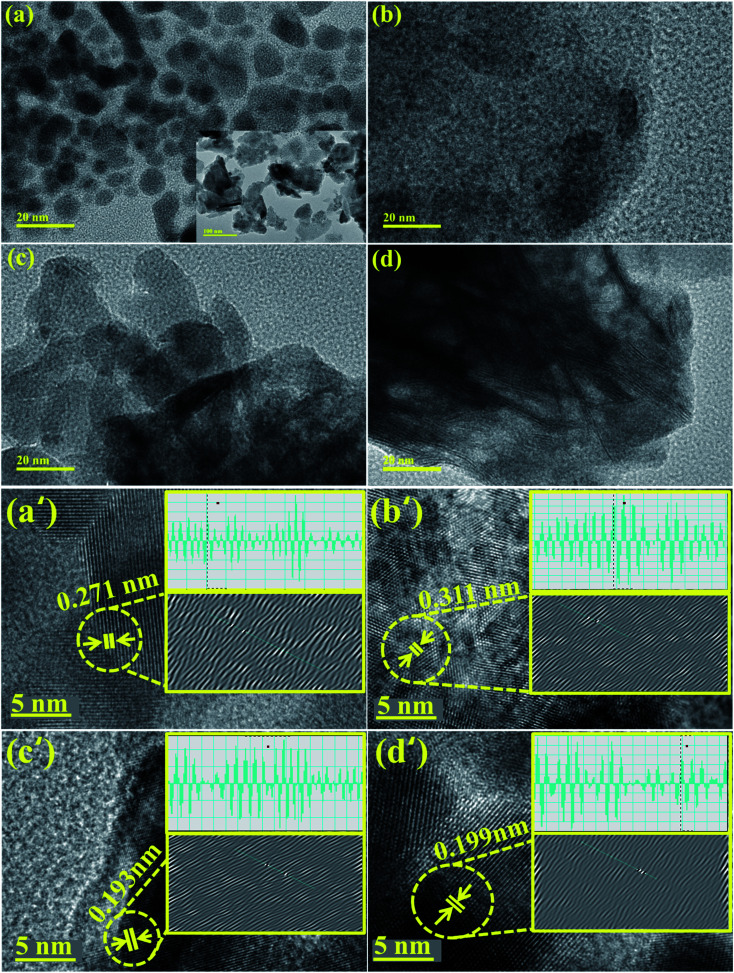
(a–d) TEM image of Bi_2_O_3_ and PVP/CS (2%, 4%)-doped Bi_2_O_3_ and (a′–d′) *d* spacing of Bi_2_O_3_ and PVP/CS (2%, 4%)-doped Bi_2_O_3_ NSs.

TEM images confirmed the morphologies of Bi_2_O_3_ and doped Bi_2_O_3_ as illustrated in Fig. 5(a–d). The image of the control sample showed multiple morphologies including quantum dots while a few nanorods were also observed (Fig. 5(a)). Addition of PVP showed that quantum dots were covered with PVP (Fig. 5(b)). Addition of low concentration of CS to PVP/Bi_2_O_3_ resulted in agglomeration of nanorods and quantum dots, which led to the formation of nanoclusters (Fig. 5(c)). Upon higher amount of CS addition, agglomeration increased with the significant nanorod-type structure of CS visible (Fig. 5(d)). Additionally, interlayer *d*-spacing was calculated from HRTEM images using Gatan software (Fig. 5(a′–d′)). Bi_2_O_3_ and PVP/CS (2%, 4%)-doped Bi_2_O_3_ NS *d*-spacing values were found to be 0.271 nm, 0.311 nm, 0.193 nm, and 0.199 nm, which are well compatible with the XRD results.

**Fig. 6:**
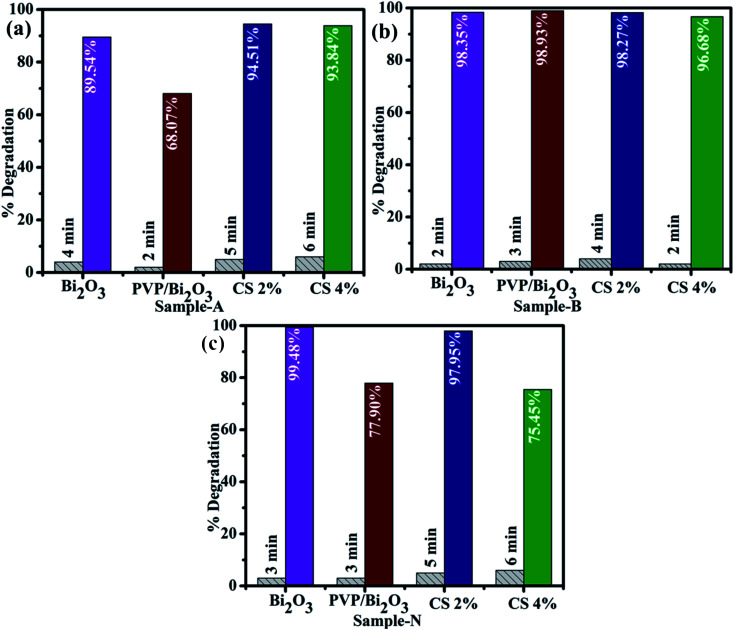
Catalytic activity of dopant free and PVP/CS (2%, 4%)-doped Bi_2_O_3_ NSs in (a) acidic medium (b) basic medium and (c) neutral medium.

Catalytic activities of pure and PVP/CS (2%, 4%)-doped Bi_2_O_3_ NSs with NaBH_4_ for MB degradation under acidic, neutral, and basic conditions were investigated using a UV-vis spectrophotometer. Dye sludge is frequently released at various pH levels; the rate of degradation is influenced by the pH solution and affects nano-catalysts that have been synthesized. Undoped and PVP/CS (2%, 4%)-doped Bi_2_O_3_ nanomaterials showed the maximum degradation of 99.48%, 77.90, 97.95%, and 75.54% in neutral (pH = 7), 98.35%, 98.93%, 98.27% and 96.68% in basic (pH = 12), and 89.54%, 68.07%, 94.51% and 93.84% in acidic (pH = 4) media (Fig. 6(a–c)). In all media, PVP/CS (2%)-doped Bi_2_O_3_ demonstrated the highest catalytic activity. The surface area crystallite size and shape of the nano-catalyst substantially influence CA. On doping with CS, variation in the dye degradation was observed, which is attributed to the presence of more active sites provided by catalyst's large surface area which results in high catalytic efficiency. In addition, the surface area is generally large, but the influence of the nano-catalyst is limited due to micro-porosity, which inhibits the reactants from diffusing to the catalyst surface.^87^ Furthermore, a slight difference between an acidic and basic medium is ascribed to increased electrostatic attraction between MB^+^, a positively charged dye and the catalyst which is negatively charged. The nanocatalyst surface in the basic medium tends to acquire a negative charge while absorption of cationic adsorbate species in acidic media is hindered by the catalyst's positively charged surfaces.^88,89^ The charge on the catalyst surface grew progressively negative as pH increased; enhancing the adsorption behavior of cationic dyes on Bi_2_O_3_ and PVP/CS (2%, 4%)-doped Bi_2_O_3_ nano-catalysts.

**Fig. 7:**
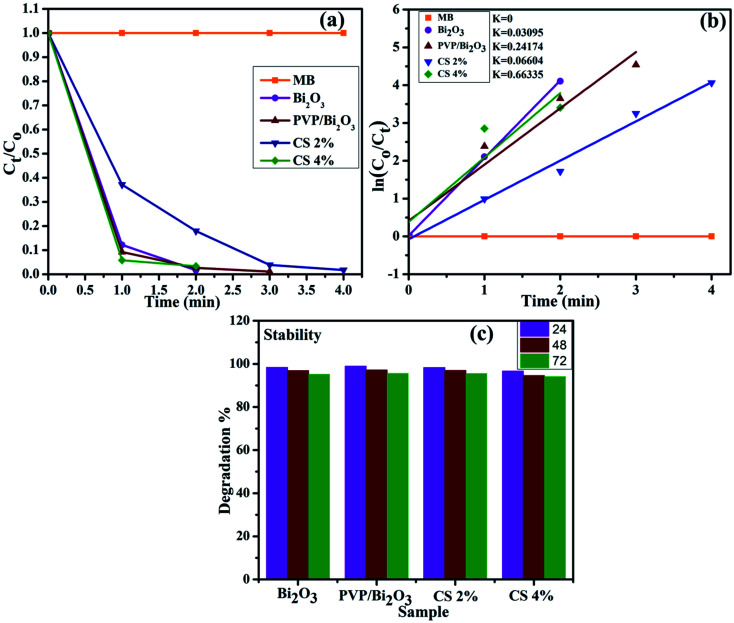
(a) Plot of the concentration ratio (*C*_*t*_/*C*_0_) *versus* time, (b) plot of ln(*C*_0_/*C*_*t*_) *versus* time spectra for dye reduction and (c) stability of the catalyst in the basic medium.

The large surface area of PVP/CS-doped Bi_2_O_3_ NSs resulted in enhanced catalytic activity. Consequently, the catalytic degradation of MB by PVP/CS (2%)-doped Bi_2_O_3_ NSs is significantly improved, and the dye is effectively degraded Fig. 7(a). The rate constants (*k*) have been calculated for catalytic degradation kinetics by measuring slopes of ln(*C*_0_/*C*_*t*_) against time. Degradation rate constant *k* for undoped and doped Bi_2_O_3_ NSs was calculated to be 0.03095, 0.24174, 0.06604 and 0.66335 min^−1^, respectively Fig. 7(b).

Investigating the stability of the nano-catalyst is of economic importance. As mentioned earlier, catalytic activity in the basic medium exhibits excellent dye degradation results. Therefore, the stability of the catalyst in the basic medium was investigated by allowing the experiment to stay for at least 72 hour in order to examine whether the reduction of dye as observed in the presence of the nanocatalyst is stable or not. In this case, the degraded dye was kept in the dark and the degradation was monitored using absorption spectra obtained through a UV-vis spectrophotometer every 24 hours, as shown in Fig. 7(c). The obtained results indicate that no loss of degradation occurred under stable conditions for 72 h. Degradation was observed to be in its fairly original form which affirms the stability of the catalyst.

**Fig. 8:**
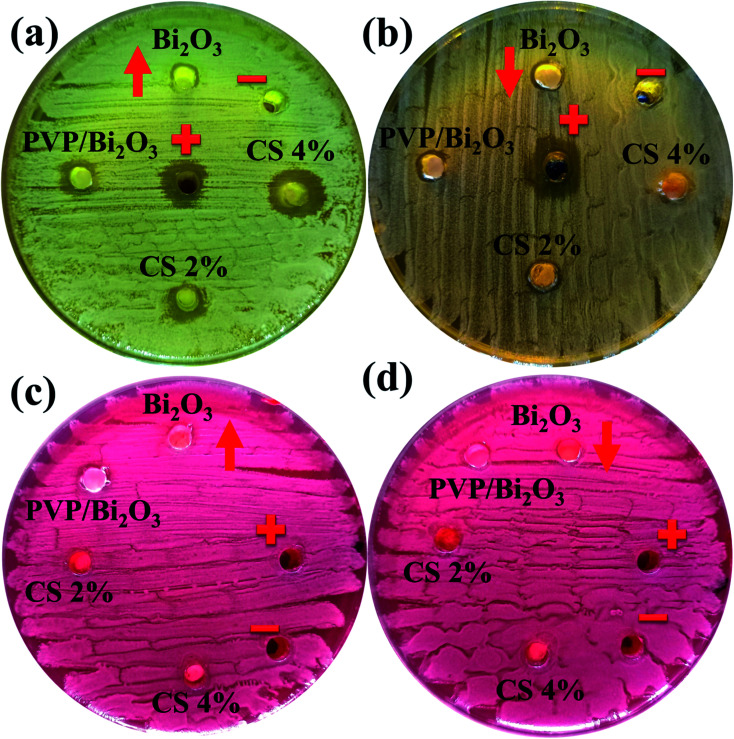
*In vitro* antimicrobial activity of the prepared NSs against (a) *S. Aureus* at high dose (b) *S. Aureus* at low dose (c) *E. coli* at high dose and (d) *E. coli* at low dose.

**Fig. 9:**
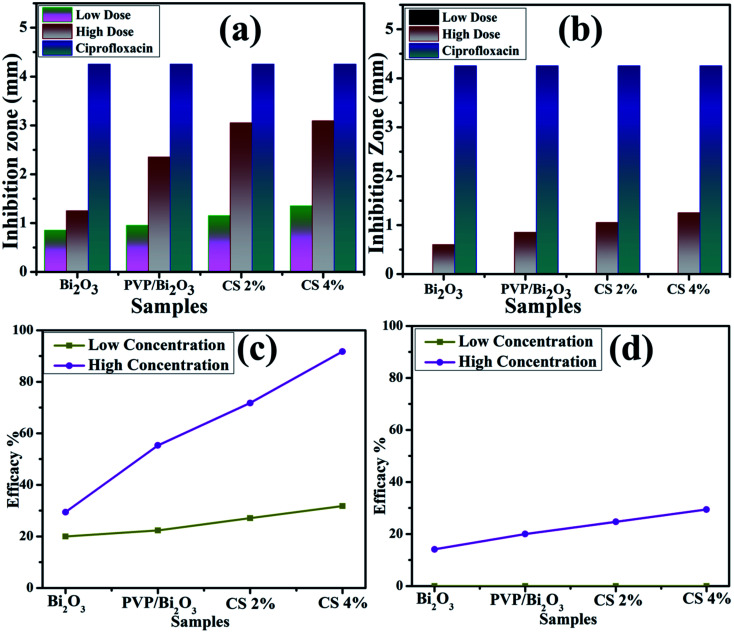
Graphical representation of antimicrobial activity of (a) *S. Aureus* and (b) *E. coli*, and efficacy of (c) *S. Aureus* and (b) *E. coli* pathogens.

Fig. 8 represents bacterial activity of doped and undoped Bi_2_O_3_ NSs which is summarized in Table 1. Comparison to the *E. coli* results reveal that doped Bi_2_O_3_ has improved bactericidal synergism and activity against *S. aureus*. The inhibition zone was recorded from (0.85–1.35) to (1.25–3.09) in *S. aureus* at low and high doses and (0.60–1.25) in *E. coli* at high dose as shown in Fig. 9(a and b). All concentrations of *E. coli* at low dose exhibited zero efficacies as shown in Fig. 9(d). A negligible efficiency was shown by Bi_2_O_3_ for *E. coli* and *S. aureus* at low and high doses respectively Fig. 9(c and d). Furthermore, inhibition zone 4.25 mm against *S. aureus* and *E. coli* for ciprofloxacin (positive control) parallel to 0 mm DIW (negative control) was recorded. Apart from this, doped Bi_2_O_3_ NSs showed substantial (*P* < 0.05) antibacterial efficacy against *S. aureus* as compared with *E. coli*. In general, cell walls of Gram negative bacteria are thicker and have a more complicated structure than Gram positive bacteria. The comparison of the present work with the literature is presented in Table 2.

**Table 1:** Antibacterial efficacy of Bi_2_O_3_ and PVP/CS (2%, 4%)-doped Bi_2_O_3_ NSs

Samples	*S. aureus*	Inhibition zone (mm)	*E. coli*	Inhibition zone (mm)
	0.5 mg/50 μL	1.0 mg/50 μL	0.5 mg/50 μL	1.0 mg/50 μL
Bi_2_O_3_	0.85	1.25	0	0.60
PVP doped Bi_2_O_3_	0.95	2.35	0	0.85
CS 2%	1.15	3.05	0	1.05
CS 4%	1.35	3.09	0	1.25
Ciprofloxacin	4.25	4.25	4.25	4.25
DIW	0	0	0	0

**Table 2:** Literature comparison of antibacterial activity of the synthesized NSs with the present study

Nano-catalyst	Synthesis route	Antibacterial activity	Outcome	References
Bi_2_O_3_ nanoparticles	Pulsed laser ablation	0% against *E. coli*	No effect at different concentrations	37
Bi_2_O_3_ nanoparticles	Bacillus licheniformis on methicillin-resistant	16% against *S. aureus*	—	38
Bi_2_O_3_ nanoparticles	Green synthesis	2 mm for *E. coli* and 1 mm for *S. aureus* in 10 mg mL^−1^ concentration	Minor enhancement in the inhibition zone on increasing the concentration	39
RGO-Bi_2_O_3_ nanocomposite	Solvothermal method	6.5 mm (100 g mL^−1^) against *E. coli*	Increase in concentration of RGO-Bi_2_O_3_ leads to higher toxicity	40
Chitosan biopolymer-functionalized zinc-doped bismuth oxide nano needle	Ultrasound-assisted chemical precipitation method	14 and 15 mm against *E. coli* and *S. aureus* respectively in 200 mg mL^−1^ concentration	—	41
Bi_2_O_3_ nanostructures	Co-precipitation	1.25 mm, 0.60 for *S. aureus* and *E. coli* respectively in 1.0 mg/50 μL concentration	By addition of dopants the antibacterial activity gradually increased	Present work

**Fig. 10:**
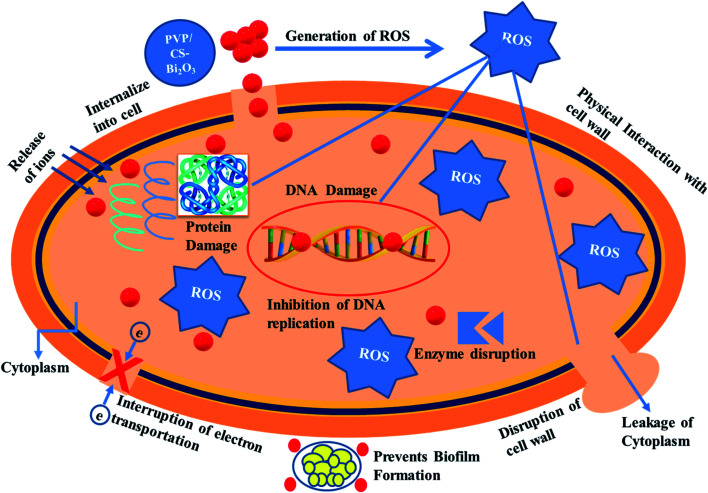
Schematic mechanism for antimicrobial activity of the prepared NSs.

Nanomaterials produce oxidative stress that is directly proportional to their concentration, shape and size. The particle size and concentration affect antibacterial activity. The size of the material has an inverse relationship with the antimicrobial efficacy.^90^ Small sized particles produce more reactive oxygen species (ROS) causing cytoplasmic components to extrude and kill bacteria by harmful microorganism membrane implant.^91,92^ Sufficient distribution of Bi^3+^ inside bacterial cells increases its antimicrobial activity as it destroys bacterial membrane stability and inhibits biofilm formation as shown in Fig. 10.^91^

## 4 Conclusion

In the present work, Bi_2_O_3_ and PVP/CS-doped-Bi_2_O_3_ NSs were successfully synthesized to achieve an improved bactericidal and catalytic activity. Among all the prepared samples, CS doping in PVP-Bi_2_O_3_ with 2% and 4% concentrations showed effective catalytic and antimicrobial activities, respectively. In view of the experimental results, Bi_2_O_3_ exhibited a monoclinic structure with varying crystallite sizes (69.5 nm, 17.5 nm, 58.4 nm, and 26.25 nm) upon PVP and CS doping. The peak shifts observed towards lower wavelength revealed with FTIR confirmed the presence of dopants while a significant peak was observed at 540 cm^−1^ for Bi_2_O_3_. Aggregated quantum dot morphology of Bi_2_O_3_ was observed while with the addition of PVP, a layer was formed on quantum dots and a nanocluster was observed upon CS doping. Additionally, nanocluster formation was recorded with an increasing amount of CS, all of which was confirmed with TEM images. The interlayer *d*-spacing (0.271 nm, 0.311 nm, 0.193 nm, and 0.199 nm) was calculated with HR-TEM images showing good agreement with XRD. Optical properties and band gap (4.18 eV, 4.27 eV, 4.09, and 4.13 eV) results were obtained through a UV-vis spectrophotometer. PL spectroscopy revealed a lower peak intensity upon doping with PVP, indicating the lower charge to hole recombination rate, whereas peak intensity was increased for different concentrations of CS, showing an enhanced charge to hole recombination. In conclusion, Bi_2_O_3_ doped NSs with natural polymers were found to be ecologically friendly, low-cost and effective against pathogens and catalytic dye degradation.

## Abbreviations

(Bi_2_O_3_): Bismuth oxide
(PVP): Polyvinylpyrrolidone
(CS): Chitosan
(CA): Catalytic activity
(XRD): X-ray diffraction
(NSs): Nanostructures
(SEM): Scanning electron microscopy
(HRTEM): High resolution transmission electron microscopy
(EDS): Energy dispersive X-ray spectroscopy
(SAED): Selected area electron diffraction
(FTIR): Fourier transform infrared spectroscopy
(PL): Photoluminescence
(MB): Methylene blue

## Data availability

Data is available on suitable demand.

## Conflicts of interest

The authors declare no conflict of interest.

## Supplementary Material
